# Interaction of Gold With Red Blood Cells

**DOI:** 10.1155/MBD.1994.517

**Published:** 1994

**Authors:** Yafei Zhang, Evelyn V. Hess, Keith G. Pryhuber, John G. Dorsey, Katherine Tepperman, R. C. Elder

**Affiliations:** 1 Biomedical Chemistry Research Center Departments of Chemistry and Biological Sciences and Division of Immunology University of Cincinnati Cincinnati OH 45221-0172 USA


### INTERACTION OF GOLD WITH RED BLOOD CELLS

Yafei Zhang, Evelyn V. Hess, Keith G. Pryhuber, John G. Dorsey, Katherine Tepperman and R. C. Elder

Biomedical Chemistry Research Center, Departments of Chemistry and Biological Sciences and Division of Immunology, University of Cincinnati, Cincinnati, OH 45221-0172, USA

Gold compounds are among the few therapeutic agents that can cause remission of aggressive rheumatoid arthritis. Although the mechanism for their toxicity and efficacy is unknown, gold levels in blood have been emphasized recently. In this report, we describe a systematic method to study the gold interaction with red blood cells and its distribution in the cell. Flow injection analysis (FIA) and high performance liquid chromatography HPLC) with gold-specific inductively coupled plasma-mass spectrometry (ICP-MS) detection are used.

Blood samples from patients being treated with Myochrysine were used to monitor the gold levels in red blood cells. Using FIA and ICP-MS , we have observed that different patients on the same regimen of drug injections differ significantly in the distribution of gold in their blood. Although the sera of these patients generally exhibit similar levels, the gold levels in their red blood cells are very different, which may reflect the fact that different patients have different responses to the drugs. The gold levels in serum can be as high as 2.5 ppm and reach "equilibrium" values after about 30 days therapy. Gold levels in red blood cells are quite variable, 400 ppb for one patient and 2.4 ppm for another. To determine the intracellular binding sites for gold, high and low molecular weight fractions were studied using HPLC-ICP-MS. Size exclusion separations show that the majority of the protein-bound gold is not bound to hemoglobin, but to a high molecular weight fraction of approximately 300,000 Da. Low molecular weight species in the red blood cell lysates were isolated by using a centrifilter with a molecular weight cutoff of 10, 000 Da, then separated by C18 reversed-phase ion pairing chromatography. One of the gold species present in the cell lysates is dicyanogold(I), which has also been identified as a free ion in patient sera. This dicyanogold(I) represents 4% of the total gold in low molecular weight, gold-containing species in the cells. A second gold species has been tentatively identified as a bis(glutathione)gold(I) complex.

Studies on the interaction of dicyanogold(I) with red blood cells were carried out by incubating the cells from naive blood with this compound in vitro. When whole blood or washed red blood cells are incubated with dicyanogold(I) most of the gold is rapidly taken up by the cells (>90% of the gold ). Gold bound to low molecular weight species represents about 3% of the total gold in the lysate solution. Auranofin shows similar uptake by red blood cells. This is in contrast to Myochrysine incubations in which only 5% of the gold is found in cell lysates. The mechanism for uptake of dicyanogold(I) is not known. One possibility is that dicyanogold(I) is taken up through the anion channel of the red blood cell. We tested this idea by using a specific inhibitor of the anion channel, 4,4'-diisothiocyanatostilbene-2,2'-DL-sulfonic acid, DIDS. A concentration of DIDS up to 3mM, sufficient to block anion uptake in red blood cells, had no effect on the uptake of gold after incubation of the red cells with dicyanogold(I) This indicates that dicyanogold(I) does not enter red blood cells through the anion channel. Another mechanism which has been proposed for uptake of gold complexes utilizes the so-called sulfhydryl shuttle, in which gold(I) complexes undergo ligand exchange to bind to sulfhydryl groups in or on the membrane. In a series of exchange reactions, the gold(I) passes through the membrane where it is then released into the cytosol. Since hydrogen cyanide would be released in the reaction of dicyanogold(I) with surface sulfhydryls, excess cyanide should repress this reaction. We therefore incubated red blood cells with sodium cyanide, followed by dicyanogold(I) . The results show that excess cyanide significantly inhibits the uptake of gold, which suggests that the loss of cyanide from dicyanogold(I) is important in red blood cell uptake of gold from dicyanogold(I) .This result is consistent with the sulfhydryl shuttle. Experiments are in progress to test this model further.

